# Supplementary material for: The discovery of novel noncoding RNAs in 50 bacterial genomes
Source: Nucleic Acids Res. 2024 Apr 22;52(9):5152–65. doi: 10.1093/nar/gkae248 (PMC11109978; doi:10.1093/nar/gkae248)
Supplement: gkae248_Supplemental_Files [file gkae248_supplemental_files.zip › Narunsky 50 Genomes RNA Motifs Files.zip!/Publication/55-Hydrogenobaculum_sp_Y04AAS1/Figures/LRC-55-2-NC_011126_1_IGR_19_779377_779488.sto.pdf]

LRC-55-2-NC_011126_1_IGR_19_779377_779488.sto.r2r

5´-AACAUCACCUCCUUAAAUUGGUGCUGAUAUUAUACCACAAUUYGAGCUAUUAUGCUUAAGUAUR●AU○○○○R●GUUYAYU●YAU●GY●A○○○○○○○○○○○○○○○○○○○○○
